# Calibration of the Hall Measurement System for a 6-DOF Precision Stage Using Self-Adaptive Hybrid TLBO

**DOI:** 10.3390/s16060872

**Published:** 2016-06-14

**Authors:** Zhenyu Chen, Yang Liu, Zhenxian Fu, Shenmin Song, Jiubin Tan

**Affiliations:** 1 Department of Control Science and Engineering, Harbin Institute of Technology, Harbin 150001, China; hitczy@163.com (Z.C.); gaea@hit.edu.cn (Z.F.); songshenmin@hit.edu.cn (S.S.); 2 Ultra-Precision Optoelectronic Instrumentation Engineering Centre, Harbin Institute of Technology, Harbin 150001, China; jbtan@hit.edu.cn

**Keywords:** hall sensor, multi-DOF precision stage, TLBO algorithm, measurement system modeling

## Abstract

To determine the planar motion of a 6-DOF precision stage, a measurement system based on three Hall sensors is adopted to obtain the X, Y, Rz motions of the stage. The machining and assembly errors in the actual mechanical system, which are difficult to measure directly, cause the parameters in the model of the Hall measurement system to deviate from their designed values. Additionally, the vertical movement of the stage will render the measurement model nonlinear. To guarantee the accuracy of the measurement, the parameters in the measurement model should be estimated and the nonlinearity compensated. In this paper, a novel approach based on self-adaptive hybrid TLBO (teaching-learning-based-optimization) is proposed to estimate the parameters in the Hall measurement model. The influences of zero deviations and vertical movements on the measurement accuracy are analyzed and compensated. The effectiveness of the proposed method is validated by experimental results obtained on a 6-DOF precision stage. Thanks to parameter estimation and calibration, the measurement error of the Hall sensor array is reduced to 6 micrometers.

## 1. Introduction

Precision motion stage is the kernel part of an ultra-precision system which has been playing a critical role in Integrated Circuit (IC) manufacturing, optical components production, Liquid Crystal Display (LCD) fabrication, IC encapsulation, *etc*. Increased demand is putting stricter requirements on the precision stage, especially on the accuracy of its multiple degree-of-freedoms (DOFs) movement. To achieve high precision positioning of the stage, a sufficiently accurate measurement system is indispensable, for the measurement error in the feedback channel of the control loop will directly affect the system performance and cannot be suppressed by the controller in the forward channel [1]. Since the 6-DOF displacements of a stage cannot be measured by a single sensor, a measurement system composed of multiple sensors is necessary. Mura measured a 6-DOF Stewart platform stage by constructing a sensor array, composed of wire extensometers or MEMS displacement sensors [2,3]. By solving the kinematic equations, the 6-DOF moving information could be estimated. Similarly, Allred used a simplified Stewart platform stage to measure the helicopter blade [4]. Kim made use of some cooperative targets to design a measurement coordinate system, and the system parameters were determined by optimization. Then, the 6-DOF displacements could be calculated by coordinate transform [5]. However, these methods are not effective for a non-cooperative and floating stage. As noncontact sensors [6,7], Hall sensors are widely used in various industrial applications, including position, velocity and magnetic fields measurement [8,9,10,11,12,13]. Also, it is shown to be insensitive to harsh environments. For measurement of a multi-DOF stage using Hall sensor, Zhao obtained an elliptic fitting function of the magnetic field for compensation, in the case that the height between the hall probe and permanent magnet is fixed [14]. To estimate the maglev stage in planar DOFs, Kawato adopted two-axis Hall-effect sensors, constructed a model of magnetic field distribution according to the Halbach magnet matrix, and developed a Gaussian least-squares differential-correction (GLSDC) algorithm [15,16]. Sun established the model of the magnetic field and displacement using finite element analysis, and presented a 3-DOF measurement algorithm based on symmetry and linearization [17].

Note that the study in reference [14,15,16,17] is based on the assumption that the distance between the Hall probe and the permanent magnetic is fixed which, however, could not be guaranteed in practice. The slope of the Hall sensor output would vary due to the change of magnetic flux. According to [14], a variation of 1 mm in distance will cause a considerable change in the output slope, and a large error will be introduced to the Hall measurement system if only the designed parameters are employed and the influence of the relative distance variation is not calibrated.

In practice, the measurements of the precision stage by the array of Hall sensors are coupled, and should be decoupled before being utilized. However, the decoupling is subject to errors in its decoupling matrix caused by the uncertainty of mechanical parameters of the precision stage, since these parameters inevitably deviate from their designed values due to machining and assembling errors. These errors will degrade the positioning accuracy of the nano-or-micro-precision stage.

Since such mechanical parameters are hard to directly measure due to limitation of space and measurement principle, a “soft sensor” method is proposed. The so-called soft sensor technology makes use of the mathematical relationship between parameters that are directly measurable and those that are not, obtaining the concerned parameters by numerical calculation based on optimal approximating theory [18]. After nearly 40 years of development, the soft measurement technology has been widely employed by process control systems in the chemical industry, metallurgy, biology, *etc*. Up to now, modeling methods for soft sensors fall into two major categories: the mechanism based and the data driven. Using the mechanism based approach, Prasad [19] obtained the model of a styrene polymerization process, and then estimated the average molecular weight of the polymer performance indexes using an extended Kalman filter. By the data driven method, Zamprogna performed principal component analysis for a batch distillation process using the regression process model and the temperature measurement [20]; Gonzaga measured the viscosity of polymer using a feed-forward artificial neural network model [21]; Liu solved the melt viscosity measurement problem in a polymer extrusion process using a nonlinear observer-based finite impulse response model, with the temperature and screw speed as its input [22]. In mechatronic systems, the concept of soft sensors has been rarely mentioned. However, some parameter estimation methods based on the prior model structures, such as Luenberger observer, Kalman filter, and adaptive observer, have also been well developed and applied in practice. Elmas adopted a full-order Luenberger observer to estimate the rotator position of a switched reluctance machine, with satisfactory effect [23]. Szabat estimated the internal states of a string-linked double mass system using a fuzzy Luenberger observer, to control the driven system [24]. Sim designed a novel Kalman filter to estimate the parameters of PMSM online [25]. Rajamani applied an adaptive observer in a vehicle suspension system [26]. Lim proposed an online frequency parameter estimation method based on statistics, to measure the rigidity and damping coefficients in the electromagnet suspension system [27].

The measurement model of the Hall sensor array, which is a mechatronic system, could be established based on mechanism analysis, which contains mechanical assembly errors and some unknown coefficients. As the relationship between these immeasurable parameters and the measurable ones is of high dimension, high order and nonlinear, the aforementioned state space-based methods are no longer suitable to this problem.

Meta-heuristic methods, inspired by natural activities, provide another approach to parameter estimation. As long as the fitness function is determined, they could be easily applied in practice without additional constraints. Owing to their simplicity, various kinds of meta-heuristic algorithms have been proposed and effectively applied in many fields, such as mechanical design and trajectory planning of robotics. The most popular meta-heuristic methods include Genetic Algorithm (GA) [28], Differential Evolution (DE) [29], Particle swarm optimization (PSO) [30], and Teaching-Learning-Based Optimization (TLBO) [31]. TLBO is newly proposed and is used for optimizing constrained mechanical design problems. Compared with other heuristic methods, the biggest advantage of TLBO is that fewer parameters are to be adjusted, with only the population size and maximum iterations to be determined [32,33,34]. However, the TLBO is prone to prematurity for some practical problems [35]. To address this issue, Ghasemi combined the traditional TLBO with the Gaussian-Based TLBO (GBTLBO), balancing the convergence rate and the solution quality [36]. In this paper, the GBTLBO is further improved using an adaptive updating strategy for the parameter *p*, the mutation probability. Applying this novel self-adaptive hybrid TLBO to the parameter estimation for the Hall measurement system, the measurement accuracy could be improved.

The rest of this paper is organized as follows: Section 2 describes the configuration of the investigated precision stage. Section 3 establishes the measurement and the calibration model of the Hall sensor array, which contains a mechanical assembly error. Section 4 proposes a self-adaptive hybrid TLBO method for estimating parameters in the aforementioned model. Then, Section 5 conducts experiments and analyzes the experimental results in different situations. Section 6 concludes the paper.

## 2. System Configuration

**Figure 1:**
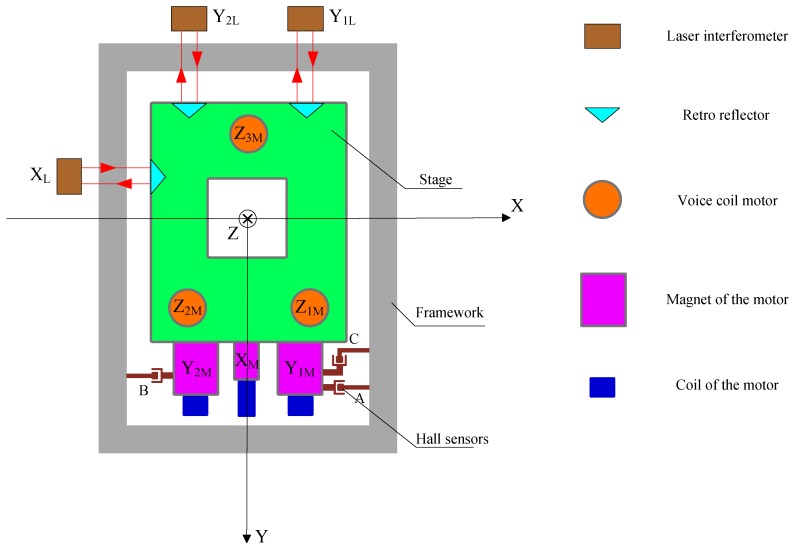
The configuration of the 6-DOF precision stage.

The structure of the 6-DOF precision stage investigated in this paper is shown in Figure 1, whose coordinate system follows the right-hand rule.

The stage is driven by six voice coil motors. Three of them, $Z_{1M}$, $Z_{2M}$, $Z_{3M}$, are in the vertical direction, constituting an isosceles triangle. Driven by these motors, the stage could realize translation in *Z* axis and rotation around *X*, *Y* axis. The driving centers of $Z_{1M}$ and $Z_{2M}$ are located in the first and second quadrants of the coordinate system respectively, symmetric to the *Y* axis. The driving center of $Z_{3M}$ is on the negative *Y* axis. The forces of these motors are parallel to the *Z* axis and have the same positive direction. In the horizontal plane, two symmetrically distributed voice coil motors, $Y_{1M}$ and $Y_{2M}$, provide driving force in Y direction. While motor $X_{M}$, located in the middle between the two Y-direction motors, provides the driving force in X direction. The driving point of motor $X_{M}$ is located on the positive *Y* axis, taking positive X direction as driving direction. The driving points of $Y_{1M}$ and $Y_{2M}$ are located in the first and second quadrants, symmetric to the *Y* axis, taking the positive direction of *Y* axis as driving direction. Since the driving points of these horizontal linear motors do not coincide, the stage can achieve horizontal rotation and translation.

**Figure 2:**
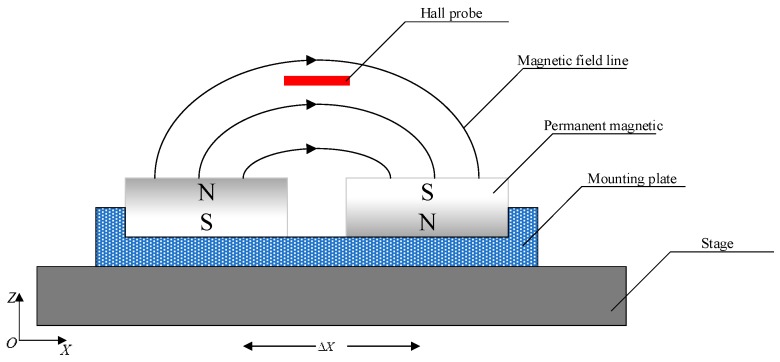
The structure of the designed Hall sensor.

**Figure 3:**
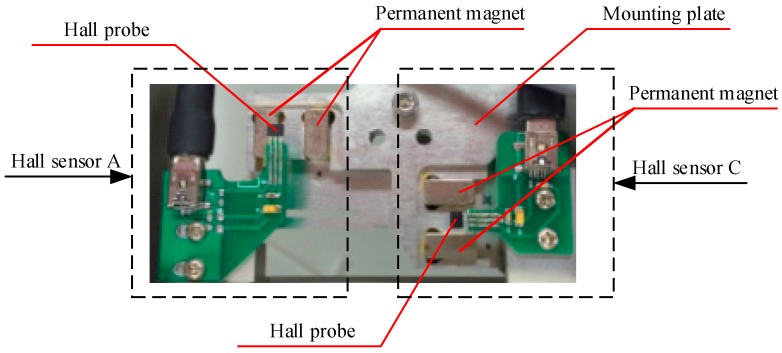
Part of Hall sensor array.

The motion of the stage is measured by the hall sensor array and the capacitance sensor. Three hall sensors, A, B, C, are responsible for measuring the displacement of the stage relative to the framework in the horizontal plane. Specifically, sensors A and B measure the relative displacements in the Y direction, and sensor C in the X direction. The output voltage of a Hall sensor is in direct proportion to the magnetic flux density where the sensor is placed, and zero output is desired in the middle of its measurement range. The structure of the Hall sensor used in the stage is shown in Figure 2, in which the structure of the magnetic steel is simplified for better versatility and easier installation. The Hall probe is fixed on the stage, while the permanent magnets are installed on the mounting plate. The dimension of the magnet is 12.7 mm × 6.4 mm × 6.4 mm, and the installation mount is designed to be 43 mm × 25.5 mm × 6.5 mm. With this configuration, an arc-shape magnetic field is generated above the upper surface of the magnet. The Hall sensor array is shown in Figure 3.

**Figure 4:**
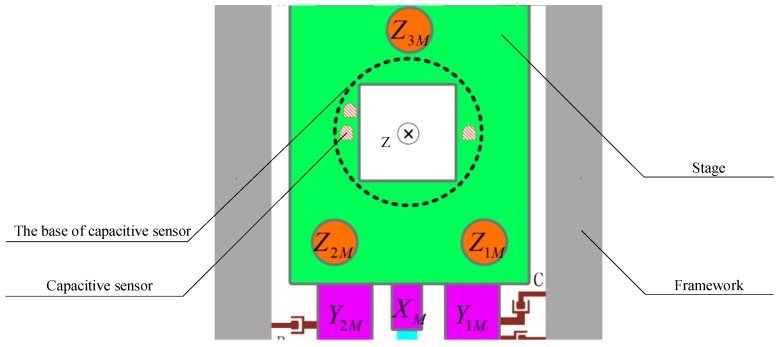
The layout of the capacitance sensors.

The vertical 3-DOF movement of the stage is measured by the capacitance sensors, whose layout is as shown in Figure 4. The probe of capacitance sensor is fixed on a disc-shaped base below the stage. Two capacitance sensors are installed on the *X* axis, symmetric to the origin, while the third capacitance sensor is located off the axis *X*. Each capacitance sensor could obtain the displacement of the probe relative to the framework, and the 3-DOF vertical movement of the stage relative to the framework can be calculated.

**Figure 5:**
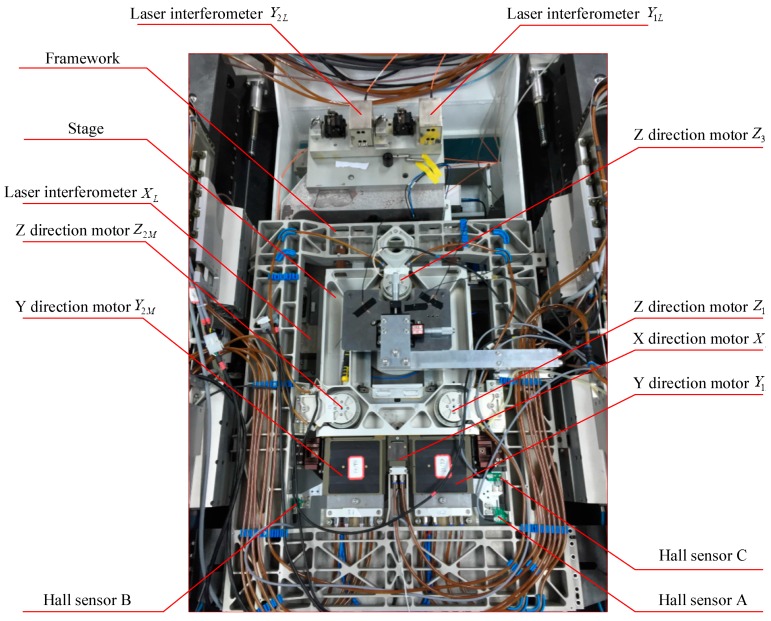
The precision stage.

To calibrate the Hall sensor array, laser interferometers are adopted to measure the planar 3-DOF motion of the stage, acting as a reference for Hall sensor array. The investigated precision stage is shown in Figure 5.

## 3. Model Establishment

### 3.1. The Decoupling Model of the Hall Sensor Array

**Figure 6:**
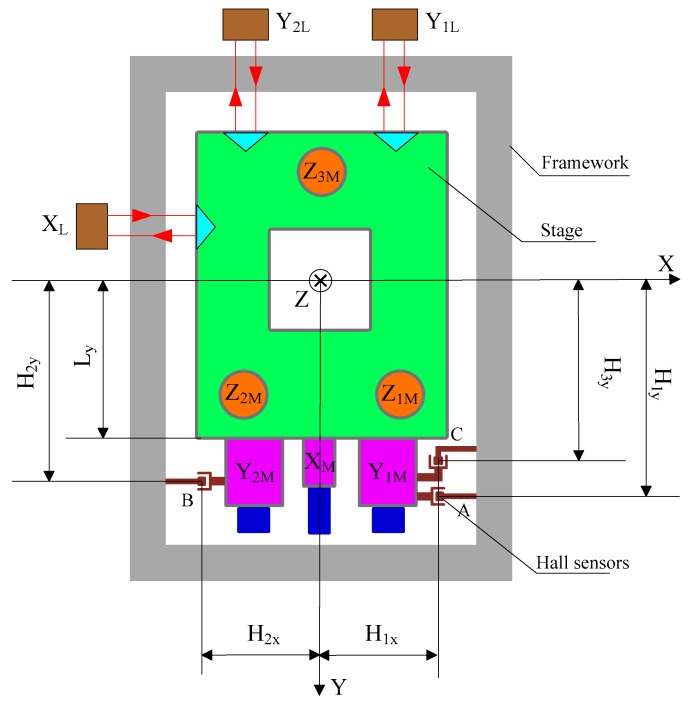
The dimension figure of the investigated stage.

To establish the decoupling model of the Hall sensor array, the structure of the precision stage marked with geometrical information is shown in Figure 6. Assume that when the stage is at its initial position, the outputs of the three Hall sensors, A, B, C, are all zero. At this position, the coordinates of the probes of the three hall sensors are $A(H_{1x},H_{1y},0)$, $\text{B}(-H_{2x},H_{2y},0)$ and $C(H_{3x},H_{3y},0)$ respectively. The movement of the stage can be decomposed into the following three scenarios: (1) firstly XY translation, then Rz rotation; (2) firstly Rz rotation, then XY translation; (3) firstly X (Y) translation, then Rz direction rotation, then Y (X) translation. The decoupling method of the sensor A under the three scenarios will be analyzed respectively. Assume that in the framework-fixed coordinate system, the stage is at its initial position at the initial moment, the movement of stage centroid in X, Y and Rz direction is Δx, Δy, ΔRz respectively.

Situation (1):

The stage moves Δx in X direction and then Δy in Y direction. Since the probe A is fixed on the framework, the relative coordinate of stage body centroid is

(1) $$\{\begin{array}{c} x_{A}{}^{\prime }=H_{1x}-\Delta x\\ y_{A}{}^{\prime }=H_{1y}-\Delta y \end{array}$$

Subsequently, the stage rotates along Rz direction, and we have

(2) $$\left[\begin{array}{c} x_{A}{}^{\prime \prime }\\ y_{A}{}^{\prime \prime } \end{array}\right]=\left[\begin{array}{cc} \cos\left(-\Delta Rz\right) & -\sin\left(-\Delta Rz\right)\\ \sin\left(-\Delta Rz\right) & \cos\left(-\Delta Rz\right) \end{array}\right]\left[\begin{array}{c} x_{A}{}^{\prime }\\ y_{A}{}^{\prime } \end{array}\right]$$

where $\left(x_{A}{}^{\prime \prime },y_{A}{}^{\prime \prime }\right)$ is the coordinates of point A in the centroid coordinates. Equation (2) can be rewritten as

(3) $$\left[\begin{array}{c} x_{A}{}^{\prime \prime }\\ y_{A}{}^{\prime \prime } \end{array}\right]=\left[\begin{array}{cc} \cos\left(\Delta Rz\right) & \sin\left(\Delta Rz\right)\\ -\sin\left(\Delta Rz\right) & \cos\left(\Delta Rz\right) \end{array}\right]\left[\begin{array}{c} x_{A}{}^{\prime }\\ y_{A}{}^{\prime } \end{array}\right]$$

The moving range of the stage is quite small, with Rz rotation within 1 mrad, and X, Y translation within 2 mm. According to the Maclaurin formula of sine and cosine function, within the moving range of the stage, it is reasonable to adopt approximations of sine and cosine functions to reduce the amount of calculation without affecting accuracy. As a result, the coordinate of point A in the centroid coordinates can be expressed as

(4) $$\left[\begin{array}{c} x_{A}{}^{\prime \prime }\\ y_{A}{}^{\prime \prime } \end{array}\right]=\left[\begin{array}{cc} 1 & \Delta Rz\\ -\Delta Rz & 1 \end{array}\right]\left[\begin{array}{c} H_{1x}-\Delta x\\ H_{1y}-\Delta y \end{array}\right]$$

Similarly, the coordinates of B and C can be also obtained as

(5) $$\left[\begin{array}{c} x_{B}{}^{\prime \prime }\\ y_{B}{}^{\prime \prime } \end{array}\right]=\left[\begin{array}{cc} 1 & \Delta Rz\\ -\Delta Rz & 1 \end{array}\right]\left[\begin{array}{c} -H_{2x}-\Delta x\\ H_{2y}-\Delta y \end{array}\right]$$

(6) $$\left[\begin{array}{c} x_{C}{}^{\prime \prime }\\ y_{C}{}^{\prime \prime } \end{array}\right]=\left[\begin{array}{cc} 1 & \Delta Rz\\ -\Delta Rz & 1 \end{array}\right]\left[\begin{array}{c} H_{3x}-\Delta x\\ H_{3y}-\Delta y \end{array}\right]$$

Furthermore, the coordinates of the Hall sensor A, B, C could be written as

(7) $$\{\begin{array}{c} \begin{array}{l} x_{A}{}^{\prime \prime }=H_{1x}-\Delta x+H_{1y}\cdot \Delta Rz-\Delta Rz\cdot \Delta y\\ y_{A}{}^{\prime \prime }=-H_{1x}\cdot \Delta Rz+\Delta Rz\cdot \Delta x+H_{1y}-\Delta y \end{array}\\ \begin{array}{l} x_{B}{}^{\prime \prime }=-H_{2x}-\Delta x+H_{2y}\cdot \Delta Rz-\Delta Rz\cdot \Delta y\\ y_{B}{}^{\prime \prime }=H_{2x}\cdot \Delta Rz+\Delta Rz\cdot \Delta x+H_{2y}-\Delta y \end{array}\\ \begin{array}{l} x_{C}{}^{\prime \prime }=H_{3x}-\Delta x+H_{3y}\cdot \Delta Rz-\Delta Rz\cdot \Delta y\\ y_{C}{}^{\prime \prime }=-H_{3x}\cdot \Delta Rz+\Delta Rz\cdot \Delta x+H_{3y}-\Delta y \end{array} \end{array}$$

Given that the moving range is so small, the quadratic terms can be ignored. Then, Equation (7) is simplified as

(8) $$\left[\begin{array}{c} \begin{array}{l} x_{A}{}^{\prime \prime }\\ y_{A}{}^{\prime \prime } \end{array}\\ \begin{array}{l} x_{B}{}^{\prime \prime }\\ y_{B}{}^{\prime \prime } \end{array}\\ \begin{array}{l} x_{C}{}^{\prime \prime }\\ y_{C}{}^{\prime \prime } \end{array} \end{array}\right]=\left[\begin{array}{ccc} -1 & 0 & H_{1y}\\ 0 & -1 & -H_{1x}\\ -1 & 0 & H_{2y}\\ 0 & -1 & H_{2x}\\ -1 & 0 & H_{3y}\\ 0 & -1 & -H_{3x} \end{array}\right]\left[\begin{array}{c} \Delta x\\ \Delta y\\ \Delta Rz \end{array}\right]+\left[\begin{array}{c} H_{1x}\\ H_{1y}\\ H_{2x}\\ H_{2y}\\ H_{3x}\\ H_{3y} \end{array}\right]$$

**Figure 7:**
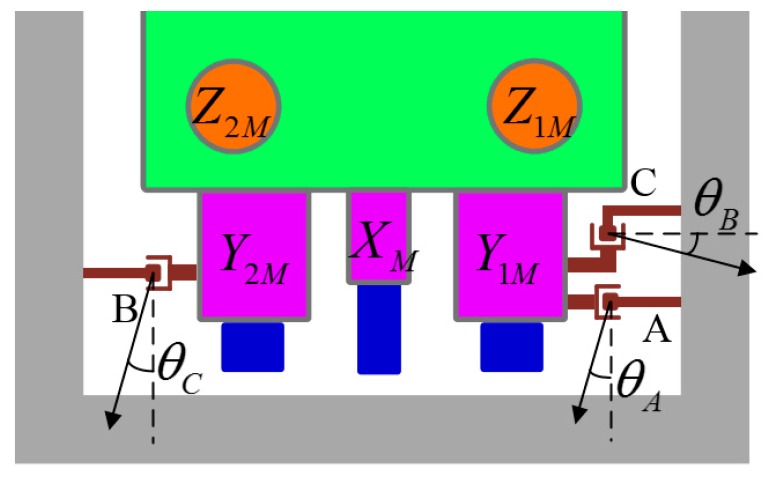
The angular assembly errors of the Hall sensor array.

As a result, the coordinates of the sensing probes in the stage centroid-origined frame could be expressed as

(9) $$\left[\begin{array}{c} \begin{array}{l} \Delta x_{A}{}^{\prime \prime }\\ \Delta y_{A}{}^{\prime \prime } \end{array}\\ \begin{array}{l} \Delta x_{B}{}^{\prime \prime }\\ \Delta y_{B}{}^{\prime \prime } \end{array}\\ \begin{array}{l} \Delta x_{C}{}^{\prime \prime }\\ \Delta y_{C}{}^{\prime \prime } \end{array} \end{array}\right]=\left[\begin{array}{ccc} 1 & 0 & -H_{1y}\\ 0 & 1 & H_{1x}\\ 1 & 0 & -H_{2y}\\ 0 & 1 & -H_{2x}\\ 1 & 0 & -H_{3y}\\ 0 & 1 & H_{3x} \end{array}\right]\left[\begin{array}{c} \Delta x\\ \Delta y\\ \Delta Rz \end{array}\right]$$

where $\Delta x_{A}{}^{\prime \prime }$, $\Delta y_{A}{}^{\prime \prime }$, $\Delta x_{B}{}^{\prime \prime }$, $\Delta y_{B}{}^{\prime \prime }$, $\Delta x_{C}{}^{\prime \prime }$, $\Delta y_{C}{}^{\prime \prime }$ denote the translational displacement of Hall sensor in stage centroid-origin. However, the sensitive axis of a Hall sensor may not be aligned with the corresponding axis of the stage centroid-origin due to installation error. The unknown angular deviations are shown in Figure 7, where $\theta_{A}$, $\theta_{B}$, $\theta_{C}$ are the angles between the sensitive axis of the Hall sensors and the corresponding coordinate axes.

By projecting the changes of coordinates of the points A, B, C in the stage centroid-origin frame onto the sensitive axis, the measurement values of the Hall sensors could be written as

(10) $$\left[\begin{array}{c} \Delta y_{A}\\ \Delta y_{B}\\ \Delta x_{C} \end{array}\right]=\left[\begin{array}{cccccc} -\sin\theta_{A} & \cos\theta_{A} & 0 & 0 & 0 & 0\\ 0 & 0 & -\sin\theta_{B} & \cos\theta_{B} & 0 & 0\\ 0 & 0 & 0 & 0 & \cos\theta_{C} & \sin\theta_{C} \end{array}\right]\left[\begin{array}{c} \begin{array}{l} \Delta x_{A}{}^{\prime \prime }\\ \Delta y_{A}{}^{\prime \prime } \end{array}\\ \begin{array}{l} \Delta x_{B}{}^{\prime \prime }\\ \Delta y_{B}{}^{\prime \prime } \end{array}\\ \begin{array}{l} \Delta x_{C}{}^{\prime \prime }\\ \Delta y_{C}{}^{\prime \prime } \end{array} \end{array}\right]$$

Combining Equations (9) and (10) yields

(11) $$\left[\begin{array}{c} \Delta y_{A}\\ \Delta y_{B}\\ \Delta x_{C} \end{array}\right]=\left[\begin{array}{ccc} -\sin\theta_{A} & \cos\theta_{A} & H_{1x}\cdot \cos\theta_{A}+H_{1y}\cdot \sin\theta_{A}\\ -\sin\theta_{B} & \cos\theta_{B} & H_{2y}\cdot \sin\theta_{B}-H_{2x}\cdot \cos\theta_{B}\\ \cos\theta_{C} & \sin\theta_{C} & H_{3x}\cdot \sin\theta_{C}-H_{3y}\cdot \cos\theta_{C} \end{array}\right]\left[\begin{array}{c} \Delta x\\ \Delta y\\ \Delta Rz \end{array}\right]$$

Solving Equation (11), the decoupling model of the horizontal 3-DOF movement relative to the framework measured by the Hall sensor array could be expressed as

(12) $$\left[\begin{array}{c} \Delta x\\ \Delta y\\ \Delta Rz \end{array}\right]={\left[\begin{array}{ccc} -\sin\theta_{A} & \cos\theta_{A} & H_{1x}\cdot \cos\theta_{A}+H_{1y}\cdot \sin\theta_{A}\\ -\sin\theta_{B} & \cos\theta_{B} & H_{2y}\cdot \sin\theta_{B}-H_{2x}\cdot \cos\theta_{B}\\ \cos\theta_{C} & \sin\theta_{C} & H_{3x}\cdot \sin\theta_{C}-H_{3y}\cdot \cos\theta_{C} \end{array}\right]}^{-1}\left[\begin{array}{c} \Delta y_{A}\\ \Delta y_{B}\\ \Delta x_{C} \end{array}\right]$$

Situations (2) and (3):

As can be seen by comparing Equations (7) and (8), the remaining terms are the linear superposition of the translational movement of the stage in X and Y direction after the quadratic terms such as ΔRz⋅Δx have been ignored. So, similarly, the decoupling models of the stage moment in situation (2) and (3) are the same as Equation (12).

In conclusion, the decoupling model of the stage movement relative to the framework could be written as Equation (12), which can guarantee the measurement accuracy and real-time performance of the decoupling process.

### 3.2. The Calibration Model of the Hall Sensor Array

According to the previous analysis, the actual distance between the Hall sensing probe and the permanent magnet is different from its designed value due to assembly error, which will change the output slope of the Hall sensor and therefore create a difference between the actual displacement and its computed value. Using Equation (12) directly as the decoupling model will introduce measurement errors and cause degradation of control precision. Therefore, the outputs of the Hall sensor array have to be calibrated.

In reference [37], by finite element simulation of a Hall sensor, Chen finds that the output in X direction of the Hall sensor is hardly influenced by the stage movement in the Y direction when the height of the sensing probe is fixed. So, in this paper, the influence of orthogonal movement on the output of the Hall sensor is ignored. Based on the previous analysis, considering the initial mechanical assembly error, the calibration model of the Hall sensor array is defined as

(13) $$\left[\begin{array}{c} y_{Ae}\\ y_{Be}\\ x_{Ce} \end{array}\right]=\left[\begin{array}{ccc} \varphi_{yA} & 0 & 0\\ 0 & \varphi_{yB} & 0\\ 0 & 0 & \varphi_{x} \end{array}\right]\left[\begin{array}{c} y_{A}\\ y_{B}\\ x_{C} \end{array}\right]$$

where $y_{A}$, $y_{B}$, $x_{C}$ denote the outputs of the Hall sensor array under designed parameters, and $y_{Ae}$, $y_{Be}$, $x_{Ce}$ denote the outputs of the Hall sensor array after calibration.

(14) $$\begin{array}{l} \varphi_{yA}=\frac{1}{\eta_{yA}}\\ \varphi_{yB}=\frac{1}{\eta_{yB}}\\ \varphi_{x}=\frac{1}{\eta_{x}} \end{array}$$

where $\eta_{yA}$, $\eta_{yB}$, $\eta_{x}$ are undetermined coefficients.

According to the analysis of Section 3.1 and Section 3.2, the measurement model of the Hall sensor array could be expressed as

(15) $$\left[\begin{array}{c} \Delta x\left(t\right)\\ \Delta y\left(t\right)\\ \Delta Rz\left(t\right) \end{array}\right]={\left[\begin{array}{ccc} -\sin\theta_{A} & \cos\theta_{A} & H_{1x}\cdot \cos\theta_{A}+H_{1y}\cdot \sin\theta_{A}\\ -\sin\theta_{B} & \cos\theta_{B} & H_{2y}\cdot \sin\theta_{B}-H_{2x}\cdot \cos\theta_{B}\\ \cos\theta_{C} & \sin\theta_{C} & H_{3x}\cdot \sin\theta_{C}-H_{3y}\cdot \cos\theta_{C} \end{array}\right]}^{-1}\left[\begin{array}{ccc} \varphi_{yA} & 0 & 0\\ 0 & \varphi_{yB} & 0\\ 0 & 0 & \varphi_{x} \end{array}\right]\left[\begin{array}{c} \Delta y_{A}\left(t\right)\\ \Delta y_{B}\left(t\right)\\ \Delta x_{C}\left(t\right) \end{array}\right]$$

## 4. Self-Adaptive Hybrid TLBO Algorithm

Teaching-learning-based optimization (TLBO) is a new meta-heuristic method, which is inspired by a teaching and learning process used in class. In this method, only population *Np* and iteration times *Gm* need to be selected before execution of the program. The whole process of TLBO can be decomposed into two phases: the teacher phase and the learner phase. In the teacher phase, the student who has the best grade (the best fitness value) is regarded as the teacher, and he will improve the grade of the whole class. The students can absorb the teacher’s knowledge to improve quickly. In the learner phase, student can select a classmate randomly from the whole class, and then learn with him after comparing fitness with each other. This phase can further improve the performance of the whole class. At the end of the iterative process, the teacher is the output of the optimization.

### 4.1. Original TLBO

#### 4.1.1. Teacher Phase

In the teacher phase, as the global best of the population, the teacher shares their knowledge to improve the performance of the whole class. However, due to the students’ different learning capabilities, the improvement of each student is different. The updated formula of the *j*-th student $St_{ij}$ is defined as

(16) $$St_{ij}^{new}=St_{ij}^{old}+r_{1}\cdot \left[T_{i}-T_{F}\cdot Mean_{i}\right]$$

where $St_{ij}^{old}$ and $St_{ij}^{new}$ denote the $St_{ij}$ before and after learning process respectively. $r_{i}$ is a random number in the range [0, 1]. $T_{i}$ is the teacher and $Mean_{i}$ is the mean at the *i*-th iteration. $T_{F}$ is a teaching factor set as either 1 or 2 presented as

(17) $$T_{F}=round\left(1+rand_{2}\left(0,1\right)\right)$$

The new learners will compete with their predecessors and replace them if a better fitness value is achieved.

#### 4.1.2. Learner Phase

In the learner phase, a student will select a partner from class randomly, and then update their status according to the following formula,

(18) $$St_{ij}^{new}=\{\begin{array}{c} St_{ij}^{old}+rand_{3}\left(St_{ik}-St_{ij}^{old}\right)\;\;if\;\;f\left(St_{ik}\right)<f\left(St_{ij}^{old}\right)\\ St_{ij}^{old}+rand_{3}\left(St_{ij}^{old}-St_{ik}\right)\;\;if\;\;f\left(St_{ij}^{old}\right)<f\left(St_{ik}\right) \end{array}$$

where $St_{ik}$ is the randomly selected student whose serial number is k. The learning process will also improve the performance of the population. Similar to the teacher phase, if the new learner is better than the old one, the old one will be replaced.

### 4.2. Self-Adaptive Hybrid TLBO

Although the TLBO is effective in many practical problems, it could not guarantee an optimal solution, especially in high dimensional, non-linear problems. To improve the optimization results, the global exploitation and partial exploration ability of the TLBO should be balanced.

Based on bare-bones PSO proposed by Kennedy [38] and bare-bones DE proposed by Omran [39], a Gaussian mutation operation is introduced to TLBO by Ghasemi [36]. The updated formula is defined as

(19) $$St_{ij}^{new}=N\left(\frac{St_{ij}^{old}+T_{i}}{2},\left|T_{i}-St_{ij}^{old}\right|\right)$$

(20) $$St_{ij}^{new}=N\left(\frac{St_{ij}^{old}+St_{ik}}{2},\left|St_{ij}^{old}-St_{ik}\right|\right)\;\;k\neq j$$

where *N* represents a Gaussian distribution.

In GBTLBO, the population is generated by Gaussian distribution, which uses the mean and distance of $St_{ij}^{old}$ and $T_{i}$($St_{ik}$) as expectation and standard deviation, respectively. In order to improve the exploration ability and avoid prematurity, a novel mutation strategy is proposed in this paper, which is expressed as follows:

(21) $$St_{ij}^{new}=St_{ij}^{old}+N\left(0,1\right)\cdot \left|T_{i}-St_{ij}^{old}\right|$$

(22) $$St_{ij}^{new}=St_{ij}^{old}+N\left(0,1\right)\cdot \left|St_{ij}^{old}-St_{ik}\right|\;\;k\neq j$$

The Gaussian distribution generated by the modified GBTLBO still use $St_{ij}^{old}$ as the expectation. However, the population would explore the neighborhood of each individual instead of converging with the teacher or an excellent student, increasing the search range and boosting exploitation ability.

Combining the fast convergence feature of TLBO and the powerful exploitation ability of CBTLBO, a hybrid style of TLBO is proposed for balancing the capabilities of exploration and exploitation, which is expressed as

(23) $$St_{ij}^{new}=\{\begin{array}{cc} St_{ij}^{old}+r_{1}\cdot \left[T_{i}-T_{F}\cdot Mean_{i}\right],\; & rand<p\\ St_{ij}^{old}+N\left(0,1\right)\cdot \left|T_{i}-St_{ij}^{old}\right|, & otherwise \end{array}$$

where rand is a random number lies in [0, 1], p is a threshold to control the mutation probability. rand obeys the uniform distribution so that p could reflect the proportion of two kinds of mutation during the evolution.

Similarly, this hybrid mutation strategy is also introduced in Learner phase, with the corresponding updated formula written as

(24) $$St_{ij}^{new}=\{\begin{array}{cc} \{\begin{array}{c} St_{ij}^{old}+rand_{3}\left(St_{ik}-St_{ij}^{old}\right)\;\;if\;\;f\left(St_{ik}\right)<f\left(St_{ij}^{old}\right)\\ St_{ij}^{old}+rand_{3}\left(St_{ij}^{old}-St_{ik}\right)\;\;if\;\;f\left(St_{ij}^{old}\right)<f\left(St_{ik}\right) \end{array},\; & rand<p\\ St_{ij}^{old}+N\left(0,1\right)\cdot \left|St_{ij}^{old}-St_{ik}\right|\;, & otherwise \end{array}$$

Obviously, the parameter p plays a critical role in the performance of the modified TLBO by affecting the converging speed and the exploration ability. So, the optimization results depend much on the selection of p. However, it is hard to determine the value of p without adequate prior knowledge. To tackle this intractable problem, a self-adaptive method is presented. Concerning self-tuning of parameters, Qin proposed a updated method for p [40], expressed as

(25) $$p=\frac{ns_{1}\cdot \left(ns_{2}+nf_{2}\right)}{ns_{2}\cdot \left(ns_{1}+nf_{1}\right)+ns_{1}\cdot \left(ns_{2}+nf_{2}\right)}$$

where *ns*_1_ and *ns*_2_ denote the numbers of individuals successfully updated by Equations (1) and (2) in Equation (26), respectively, while *nf*_1_ and *nf*_2_ denote the numbers of those not. Using this strategy, a self-adaptive differential evolution (SaDE) with two mutation modes is presented as follows [40]:

(26) $$St_{ij}^{new}=\{\begin{array}{cc} Eq.\;(1), & rand<p\\ Eq.\;(2), & otherwise \end{array}$$

The initial value of *p* is set to be 0.5. Once *p* updates, *ns*_1_, *ns*_2_, *nf*_1_ and *nf*_2_ would be reset.

However, the success ratio of mutation could not accurately reflect the performance of the updated strategy. In case of local convergence, for instance, although the individual is updated, the improvement in the cost-function is quite small as the algorithm has already lost its exploration ability. So, the successful update ratio is not a perfect criterion. To address this shortcoming, the declining ratio of the cost-function caused by the successfully mutating individuals is introduced to the updated formula. Then, *p* is updated as

(27) $$p=\frac{vs_{1}\cdot \left(ns_{2}+nf_{2}\right)}{vs_{2}\cdot \left(ns_{1}+nf_{1}\right)+vs_{1}\cdot \left(ns_{2}+nf_{2}\right)}$$

where $vs_{1}$, $vs_{2}$ denote the declining ratio of cost-function caused by Equations (1) and (2) in Equation (26), respectively, calculated as

(28) $$\{\begin{array}{c} vs_{1}=\sum \Delta f\left(St_{ij}^{new}\right),\;\;if\;\;f\left(St_{ij}^{new}\right)<f\left(St_{ij}^{old}\right)\;\;and\;\;rand<p\\ vs_{2}=\sum \Delta f\left(St_{ij}^{new}\right),\;\;if\;\;f\left(St_{ij}^{new}\right)<f\left(St_{ij}^{old}\right)\;\;and\;\;rand\geq p \end{array}$$

(29) $$\Delta f\left(St_{ij}^{new}\right)=\frac{f\left(St_{ij}^{old}\right)-f\left(St_{ij}^{new}\right)}{f\left(St_{ij}^{old}\right)}$$

**Figure 8:**
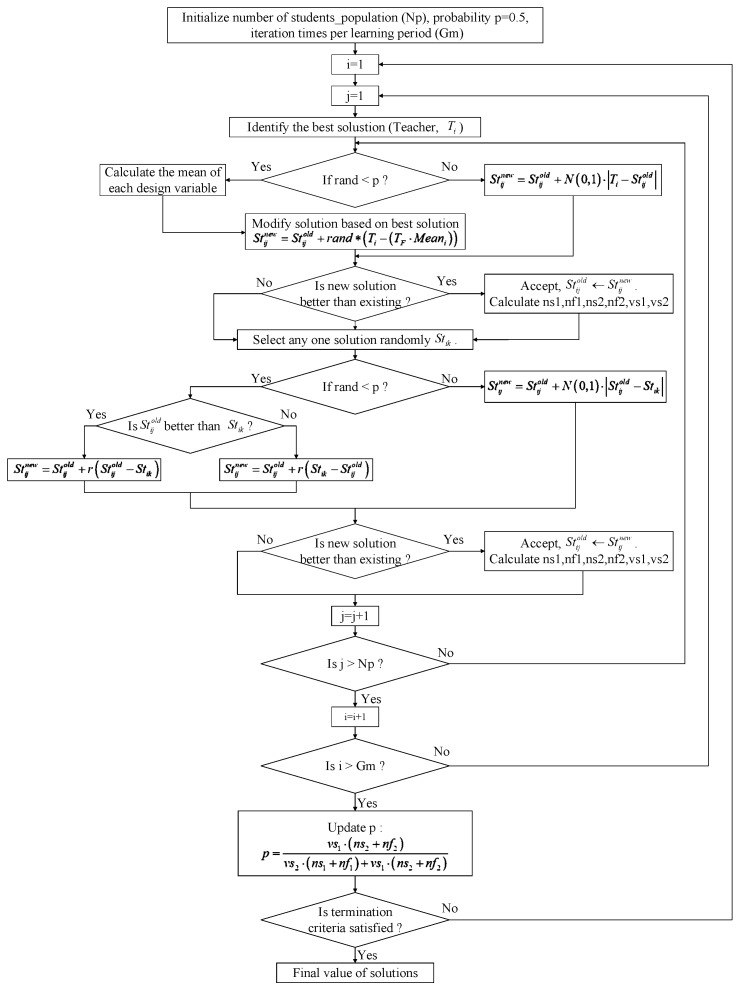
The flow chart of the self-adaptive hybrid TLBO.

This way, the updated strategy is directly related to the cost-function, and the preponderant mutation will have a greater opportunity in the next evolution iteration. The flow chart of the proposed Self-adaptive hybrid TLBO (SHTLBO) is shown in Figure 8.

## 5. Experiments and Analysis

In this section, the proposed Self-adaptive hybrid TLBO method is applied to estimate the mechanical parameters of the designed Hall measurement system for the 6-DOF precision stage illustrated in Section 2. The desired accuracy of the Hall sensor array for each axis is 5 μm, and the vertical 3-DOF movement of the stage is obtained by capacitive sensors, CAPANCDT 6500 by Micro-Epsilon, with an accuracy of 12 nm.

To calibrate the Hall sensor array, a 3-DOF laser interferometer array is adopted to provide reference. Although environmental variations, such as temperature, humidity and vibration, limited the static accuracy of the interferometer to 500 nm, it is still adequate for calibration of the Hall sensor array. To synchronize different sensors, a clock with 2 ms is designed.

To conduct the calibration experiment, the designed values of parameters in the Hall sensor array are listed in Table 1.

**Figure 9:**
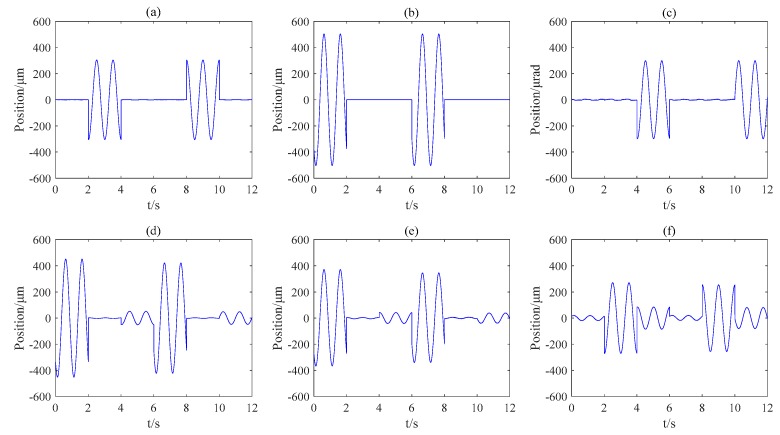
The displacements of the stage measured by the interferometers in X, Y, Rz directions (**a**–**c**) and the outputs of the Hall sensors A, B, C (**d**–**f**).

The precision stage is first controlled to stop at a fixed vertical level to avoid the disturbance induced by vertical moment. Considering the influence of vertical states on the measurements of Hall arrays, the stage is fixed at two heights: Z = −250 μm and Z = −250 μm. Then, the stage conducts the planar reset operation, providing the zero points for the 3-DOF interferometer array. After the reset, the planar motion of the stage could be measured by both the Hall sensors and the interferometers. Next, the stage is controlled to track certain trajectory with feedback from the interferometers. Then the output of the interferometers can be utilized to calibrate the Hall sensors. In this experiment, the trajectory along each axis is a sinusoidal wave: with amplitude of 300 μm and frequency of 1 Hz along *X* axis; 500 μm and 1 Hz along *Y*; 300 μrad and 1 Hz along *Z*. The corresponding outputs from the sensors are shown in Figure 9.

**Figure 10:**
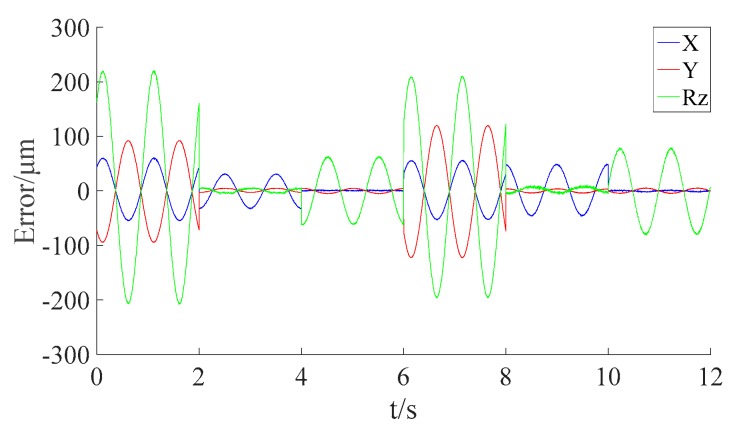
The output errors of the Hall sensor array with the designed parameters using Model (15) when the stage is on a different vertical level.

Using the designed parameters listed in Table 1 and the measurement model of the Hall sensor array built in Section 3, the output errors of Hall sensors with the designed parameters could be obtained, as shown in Figure 10.

The peak errors and the standard deviations (RMSE) of each DOF with the designed parameters are listed in Table 2.

As can be seen from Figure 10 and Table 2, the measurement accuracies of the Hall sensor array with the designed parameters fall far short of the requirements. The peak error in the translational direction is 119.9458 μm, while the peak error in the rotational direction is 221.5275 μrad. This is caused by the afore-mentioned mechanical assembly errors in the Hall sensor array. So, it is necessary to conduct the calibration. To set proper constraints on the optimization algorithm, the search ranges are given according to the mechanical design of the stage, as listed in Table 3.

**Figure 11:**
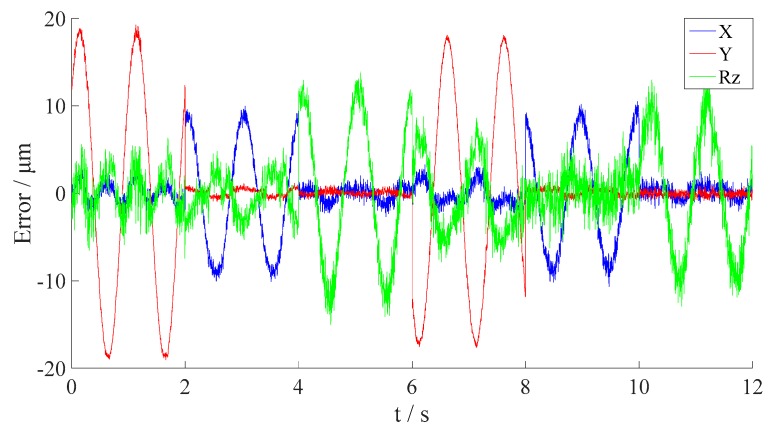
The output errors in horizontal 3-DOFs with the optimized parameters using Model (30).

In this experiment, the interferometers need to be reset using the feedback from the Hall measuring system, which contains about 5 μm measurement noise. As a result, the zero points of the interferometers vary between different rounds of movements, introducing uncertainty to the calibration system. To eliminate this offset, the initial aligning parameters should be considered in the calibration model. Therefore, Equation (15) is modified as

(30) $$\left[\begin{array}{c} \Delta x\left(t\right)\\ \Delta y\left(t\right)\\ \Delta Rz\left(t\right) \end{array}\right]={\left[\begin{array}{ccc} -\sin\theta_{A} & \cos\theta_{A} & H_{1x}\cdot \cos\theta_{A}+H_{1y}\cdot \sin\theta_{A}\\ -\sin\theta_{B} & \cos\theta_{B} & H_{2y}\cdot \sin\theta_{B}-H_{2x}\cdot \cos\theta_{B}\\ \cos\theta_{C} & \sin\theta_{C} & H_{3x}\cdot \sin\theta_{C}-H_{3y}\cdot \cos\theta_{C} \end{array}\right]}^{-1}\left[\begin{array}{ccc} \frac{1}{\eta_{yA}} & 0 & 0\\ 0 & \frac{1}{\eta_{yB}} & 0\\ 0 & 0 & \frac{1}{\eta_{x}} \end{array}\right]\left[\begin{array}{c} \Delta y_{A}\left(t\right)-\delta_{Ai}\\ \Delta y_{B}\left(t\right)-\delta_{Bi}\\ \Delta x_{C}\left(t\right)-\delta_{Ci} \end{array}\right]$$

where $\delta_{Ai}$, $\delta_{Bi}$, $\delta_{Ci}$ denote the reset errors of HALL sensors A, B and C during the *i*-th movement, respectively. The range of these variables are all [−10 μm, 10 μm]. Then, parameter estimation for the measurement Model (30) was conducted, and the corresponding results are shown in Figure 11, while the RMSE and the peak errors are listed in Table 4. To estimate the parameters, the proposed self-adaptive hybrid TLBO is adopted. The training data is composed of 100 randomly selected data from the experimental results. The setting of the optimization algorithm is: *Np* = *Gm* = 100. The stop criterion is LP>100.

As can be seen from Figure 11 and Table 4, the measurement accuracy of the Hall sensor array is improved by the parameter estimation. The peak error in translational direction is reduced to 19.2473 μm, and that in rotational direction to 15.0316 μrad, approaching the desired accuracy. But this accuracy is still far from desired value, which means that the measurement Model (30) is not accurate enough for the purpose. Therefore, the influence of vertical moments should be incorporated in the model. According to the principle of Hall measurement, the slope of the magnetic flux density sensed by the Hall probe would change with the distance between the Hall sensor and the upper surface of the permanent magnet. To accommodate this feature, a new calibration model is constructed as in Equation (31), which considers the linear combinations of vertical movements.

(31) $$\left[\begin{array}{c} \Delta x\left(t\right)\\ \Delta y\left(t\right)\\ \Delta Rz\left(t\right) \end{array}\right]=PM^{-1}\cdot \left\{\left[\begin{array}{ccc} \frac{1}{\varphi_{A}\left(t\right)} & 0 & 0\\ 0 & \frac{1}{\varphi_{B}\left(t\right)} & 0\\ 0 & 0 & \frac{1}{\varphi_{C}\left(t\right)} \end{array}\right]\left[\begin{array}{c} \Delta y_{A}\left(t\right)\\ \Delta y_{B}\left(t\right)\\ \Delta x_{C}\left(t\right) \end{array}\right]-\left[\begin{array}{ccc} \frac{1}{\varphi_{A}\left(0\right)} & 0 & 0\\ 0 & \frac{1}{\varphi_{B}\left(0\right)} & 0\\ 0 & 0 & \frac{1}{\varphi_{C}\left(0\right)} \end{array}\right]\left[\begin{array}{c} \delta_{Ai}\\ \delta_{Bi}\\ \delta_{Ci} \end{array}\right]\right\}$$

(32) $$PM=\left[\begin{array}{ccc} -\sin\theta_{A} & \cos\theta_{A} & H_{1x}\cdot \cos\theta_{A}+H_{1y}\cdot \sin\theta_{A}\\ -\sin\theta_{B} & \cos\theta_{B} & H_{2y}\cdot \sin\theta_{B}-H_{2x}\cdot \cos\theta_{B}\\ \cos\theta_{C} & \sin\theta_{C} & H_{3x}\cdot \sin\theta_{C}-H_{3y}\cdot \cos\theta_{C} \end{array}\right]$$

(33) $$\{\begin{array}{c} \varphi_{A}\left(t\right)=a_{A}\cdot Z\left(t\right)+b_{A}\cdot Rx\left(t\right)+c_{A}\cdot Ry\left(t\right)+\eta_{yA}\\ \varphi_{B}\left(t\right)=a_{B}\cdot Z\left(t\right)+b_{B}\cdot Rx\left(t\right)+c_{B}\cdot Ry\left(t\right)+\eta_{yB}\\ \varphi_{C}\left(t\right)=a_{C}\cdot Z\left(t\right)+b_{C}\cdot Rx\left(t\right)+c_{C}\cdot Ry\left(t\right)+\eta_{x} \end{array}$$

where *PM* is the matrix containing the error parameters, $\varphi_{A}\left(t\right)$, $\varphi_{B}\left(t\right)$, $\varphi_{C}\left(t\right)$ denote respectively the calibration coefficients of the three Hall sensors’ outputs which vary with the vertical moments of the stage. $\left\{a_{A},b_{A},\ldots ,c_{C}\right\}$ denote the coefficients of different DOFs, which are to be optimized. $\eta_{yA}$, $\eta_{yB}$, $\eta_{x}$ denote the calibration coefficients of the vertical zero plane; $\delta_{Ai}$, $\delta_{Bi}$, $\delta_{Ci}$ denote the zero deviations of the three Hall sensors at the *i*-th movement.

During the experiment, the vertical moving range is ±250 μm; the rotational ranges of *Rx* and *Ry* are both ±500 μrad. Considering the achievable assembly accuracy, the boundary of the TLBO searching is set to be 5000. Furthermore, the sign of the boundary could be determined according to reference [14]. Taking Hall sensor A as an example: the distance between the Hall probe and the upper surface of the permanent magnetic will increase if the stage moves in the positive vertical direction. As a result, the output slope of the Hall sensor A would decrease. Therefore, the coefficient $a_{A}$ should be negative. The signs of other coefficients can be determined likewise. Finally, the search boundary of the coefficients can be determined, as listed in Table 5.

In the experiment, the stage is set at different vertical states as listed in Table 6. Under each vertical setting, the stage is controlled in the same manner as mentioned above. Alsom the sensors’ data are sampled in the same way. Then, the parameters in Model (31) are optimized by the hybrid adaptive TLBO. The setting of the optimizing algorithm is the same as mentioned above. The training data is set to 200 points.

The estimated results of the measurement parameters are listed in Table 7, while the corresponding coefficients of zero deviations are shown in Table A1 (Appendix A).

**Figure 12:**
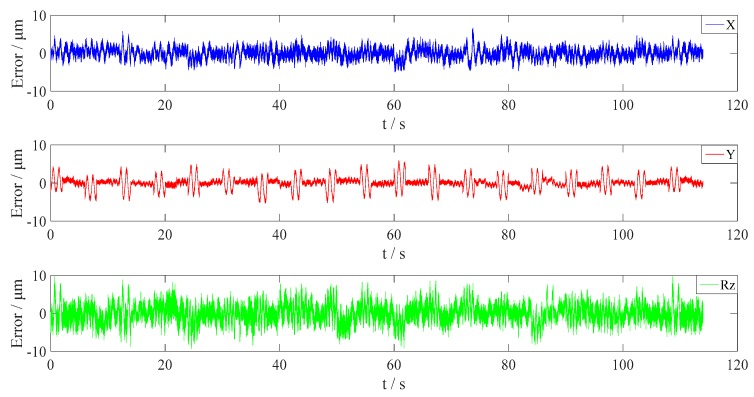
The output errors in horizontal 3-DOFs with the optimized parameters using Model (31).

Apply the estimated results to Model (31), and then validate it using all the sampling data. The output errors of X, Y and Rz are shown in Figure 12 and Table 8.

As seen from Figure 12 and Table 8, by calibration, the maximum translational and rotational errors of the Hall sensor array are reduced to 6.6368 μm and 9.63 μrad, respectively, which meets the measurement accuracy requirement.

## 6. Conclusions

In this paper, a 3-DOF measurement system based on the Hall sensor array is designed for a 6-DOF precision stage. Considering the mechanical assembly error of the Hall sensors, the measurement model and the corresponding calibration model of the Hall sensor array are established. Then, the influence of the vertical stage movements and the reset deviation of the interferometers are analyzed to ensure suitability for practical application. To estimate the related parameters in the measurement model, a self-adaptive hybrid TLBO method is proposed. The experimental results show that the measurement accuracy of the Hall sensor array could reach 6 micrometers using the proposed measurement parameter estimation algorithm.

## Figures and Tables

**Table 1:** The designed values of parameters in Hall sensor array.

**Parameter**	$H_{1x}$	$H_{2x}$	$H_{3y}$	$H_{3x}$	$H_{1y}$	$H_{2y}$
Value	0.1975 m	0.1975 m	0.36 m	0.1975 m	0.4 m	0.42 m
**Parameter**	$\eta_{yA}$	$\eta_{yB}$	$\eta_{x}$	$\theta_{1}$	$\theta_{2}$	$\theta_{3}$
Value	1	1	1	0°	0°	0°

**Table 2:** The output errors of the 3-DOF with designed values of parameters using Model (15).

DOF	X	Y	Rz
RMSE	27.8795 μm	44.1243 μm	89.2927 μrad
Peak error	61.2092 μm	119.9458 μm	221.5275 μrad

**Table 3:** The search ranges of the parameters using Model (15).

**Parameter**	$H_{1x}$	$H_{2x}$	$H_{3y}$	$H_{3x}$	$H_{1y}$	$H_{2y}$
Lower range	0.1475 m	0.1475 m	0.26 m	0.1475 m	0.3 m	0.32 m
Upper range	0.2475 m	0.2475 m	0.46 m	0.2475 m	0.5 m	0.52 m
**Parameter**	$\eta_{yA}$	$\eta_{yB}$	$\eta_{x}$	$\theta_{1}$	$\theta_{2}$	$\theta_{3}$
Lower range	0.78	0.65	0.72	−8°	−8°	−8°
Upper range	0.98	0.85	0.92	8°	8°	8°

**Table 4:** The statistics of the horizontal 3-DOFs’ outputs with optimized parameters using Model (30).

DOF	X	Y	Rz
RMSE	2.1896 μm	4.2560 μm	2.9176 μrad
Peak error	10.6451 μm	19.2473 μm	15.0316 μrad

**Table 5:** The search boundaries of the coefficients related with the vertical movements.

**Parameter**	$a_{A}$	$b_{A}$	$c_{A}$	$a_{B}$	$b_{B}$
Lower range	−5000	−5000	0	−5000	−5000
Upper range	0	0	5000	0	0
**Parameter**	$c_{B}$	$a_{C}$	$b_{C}$	$c_{C}$	
Lower range	−5000	−5000	−5000	0	
Upper range	0	0	0	5000	

**Table 6:** Vertical movement setting.

	DOF	Z/μm	Rx/μrad	Ry/μrad
NO.				
1		0	0	0
2		250	0	0
3		−250	0	0
4		150	0	0
5		−150	0	0
6		50	0	0
7		−50	0	0
8		0	500	0
9		0	−500	0
10		0	250	0
11		0	−250	0
12		0	100	0
13		0	−100	0
14		0	0	500
15		0	0	−500
16		0	0	250
17		0	0	−250
18		0	0	100
19		0	0	−100

**Table 7:** The estimated values of parameters using model (31).

**Parameter**	$H_{1x}$	$H_{2x}$	$H_{3y}$	$H_{3x}$	$H_{1y}$	$H_{2y}$
Value	0.1944 m	0.2173 m	0.3293m	0.1979 m	0.3381 m	0.3555 m
**Parameter**	$a_{A}$	$b_{A}$	$c_{A}$	$\eta_{yA}$		
Value	−188.1873	−69.7432	14.8664	0.8671		
**Parameter**	$a_{B}$	$b_{B}$	$c_{B}$	$\eta_{yB}$		
Value	−156.5783	−56.7511	−55.0764	0.7049		
**Parameter**	$a_{C}$	$b_{C}$	$c_{C}$	$\eta_{yx}$		
Value	−178.1902	−63.4133	12.7241	0.8632		
**Parameter**	$\theta_{1}$	$\theta_{2}$	$\theta_{3}$			
Value	0.0145°	0.4143°	−0.0561°			

**Table 8:** The statistics of the horizontal 3-DOFs’ outputs with optimized parameters using Model (31).

DOF	X	Y	Rz
RMSE	1.3919 μm	1.5381 μm	2.3057 μrad
Peak error	6.6368 μm	5.9375 μm	9.6300 μrad

## Appendix A

**Table A1:** The coefficients of zero deviation using model (31).

Setting	DOF	$\delta_{A}$/μm	$\delta_{B}$/μm	$\delta_{C}$/μm
Z = 0	Y	0.87	0.52	0.26
Rx = 0	X	0.27	−0.05	0.78
Ry = 0	Rz	0.63	0.29	2.31
Z = −250 μm	Y	1.32	0.33	0.45
Rx = 0	X	0.89	−0.24	0.93
Ry = 0	Rz	1.05	0.58	0.86
Z = +250 μm	Y	−0.93	1.60	1.92
Rx = 0	X	−0.82	1.23	0.39
Ry = 0	Rz	0.99	1.11	1.17
Z = −150 μm	Y	−0.33	1.90	0.12
Rx = 0	X	−1.10	−0.95	1.18
Ry = 0	Rz	0.09	0.41	−0.94
Z = +150μm	Y	0.01	2.15	1.11
Rx = 0	X	−0.48	−0.15	−1.10
Ry = 0	Rz	0.41	0.15	0.81
Z = −50 μm	Y	2.77	1.99	−2.80
Rx = 0	X	1.97	1.23	−0.09
Ry = 0	Rz	1.79	1.37	1.51
Z = +50 μm	Y	−0.66	1.27	0.07
Rx = 0	X	0.53	0.40	0.17
Ry = 0	Rz	1.91	0.48	−0.89
Z = 0	Y	−0.99	−0.44	0.11
Rx = −500 μrad	X	0.39	0.10	0.23
Ry = 0	Rz	1.11	0.93	−0.09
Z = 0	Y	1.28	1.23	−0.57
Rx = +500 μrad	X	1.09	1.05	0.09
Ry = 0	Rz	0.49	0.94	−1.47
Z = 0	Y	0.31	0.81	3.11
Rx = −250 μrad	X	−1.17	−0.54	2.38
Ry = 0	Rz	0.30	1.09	−0.79
Z = 0	Y	0.15	0.63	−0.42
Rx = +250 μrad	X	1.52	1.69	−0.01
Ry = 0	Rz	0.61	−0.54	0.61
Z = 0	Y	−0.07	1.84	0.03
Rx = −100 μrad	X	0.38	−0.17	−1.48
Ry = 0	Rz	1.71	0.28	0.46
Z = 0	Y	0.74	0.84	0.17
Rx = +100 μrad	X	0.01	−0.04	−1.16
Ry = 0	Rz	−0.04	1.03	2.93
Z = 0	Y	0.99	0.72	−4.22
Rx = 0	X	0.63	−0.11	1.10
Ry = −500 μrad	Rz	0.71	0.18	0.76
Z = 0	Y	0.39	−0.22	0.42
Rx = 0	X	1.70	1.90	−0.96
Ry = +500 μrad	Rz	0.02	0.02	0.36
Z = 0	Y	−0.40	1.51	−0.36
Rx = 0	X	−0.31	−0.97	−4.79
Ry = −250 μrad	Rz	−1.09	0.07	0.96
Z = 0	Y	−0.79	−0.16	−1.61
Rx = 0	X	2.93	1.01	−0.21
Ry = +250 μrad	Rz	−0.92	−0.10	1.66
Z = 0	Y	1.74	1.83	0.18
Rx = 0	X	1.73	0.58	0.08
Ry = −100 μrad	Rz	−0.09	0.11	0.30
Z = 0	Y	−0.71	0.24	1.09
Rx = 0	X	1.07	0.53	1.81
Ry = +100μrad	Rz	0.74	2.18	−0.82
